# Inhibition of Bacteria Associated with Wound Infection by Biocompatible Green Synthesized Gold Nanoparticles from South African Plant Extracts

**DOI:** 10.3390/nano7120417

**Published:** 2017-11-26

**Authors:** Abdulrahman M. Elbagory, Mervin Meyer, Christopher N. Cupido, Ahmed A. Hussein

**Affiliations:** 1 DST/Mintek Nanotechnology Innovation Centre, Department of Biotechnology, University of the Western Cape, Private Bag X17, Bellville 7535, South Africa; 3376881@myuwc.ac.za (A.M.E.); memeyer@uwc.ac.za (M.M.); 2 Botany Department, University of Forte Hare, Private Bag X1314, Alice 5700, South Africa; ccupido@ufh.ac.za; 3 Chemistry Department, Cape Peninsula University of Technology, P.O. Box 1906, Bellville 7535, South Africa

**Keywords:** gold nanoparticles, green nanotechnology, *Galenia africana*, *Hypoxis hemerocallidea*, antibacterial activity, Alamar blue, MTT, HRTEM

## Abstract

Unlike conventional physical and chemical methods, the biogenic synthesis of gold nanoparticles (GNPs) is considered a green and non-toxic approach to produce biocompatible GNPs that can be utilized in various biomedical applications. This can be achieved by using plant-derived phytochemicals to reduce gold salt into GNPs. Several green synthesized GNPs have been shown to have antibacterial effects, which can be applied in wound dressings to prevent wound infections. Therefore, the aim of this study is to synthesize biogenic GNPs from the South African *Galenia africana* and *Hypoxis hemerocallidea* plants extracts and evaluate their antibacterial activity, using the Alamar blue assay, against bacterial strains that are known to cause wound infections. Additionally, we investigated the toxicity of the biogenic GNPs to non-cancerous human fibroblast cells (KMST-6) using 3-[4,5-dimethylthiazol-2-yl]-2,5-diphenyl tetrazolium bromide (MTT) assay. In this paper, spherical GNPs, with particle sizes ranging from 9 to 27 nm, were synthesized and fully characterized. The GNPs from *H. hemerocallidea* exhibited antibacterial activity against all the tested bacterial strains, whereas GNPs produced from *G. africana* only exhibited antibacterial activity against *Pseudomonas aeruginosa*. The GNPs did not show any significant toxicity towards KMST-6 cells, which may suggest that these nanoparticles can be safely applied in wound dressings.

## 1. Introduction

The antibacterial potential of the metallic nanoparticles (NPs) have been under investigation to counter the increase of microbial resistance against the current antimicrobial agents [1]. Additionally, the potential application of the NPs in wound dressings to fight infections makes these NPs extremely useful in wound care. Different metals such as gold, silver, platinum, palladium, copper, aluminum, iron, and titanium have been used to synthesize NPs [2]. Gold nanoparticles (GNPs) in particular have attracted huge attention for their unique optical properties as well as their biocompatibility [1]. GNPs are included in a variety of applications such as separation science [3], optical sensors, food industry as well as space and environmental sciences [4]. GNPs have also shown potential in several biomedical applications. GNPs have been shown to destroy tumors by photothermal therapy [5]. Other biomedical applications of GNPs include gene therapy, drug delivery, DNA and RNA analysis and as antibacterial agents, etc. [6]. 

The use of environmentally toxic reagents, the production of harmful by-products and the use of expensive apparatus during conventional physical and chemical synthesis of metallic NPs hinder their exploitation in biomedical applications. Conversely, the green synthesis of metallic NPs involves the use of safe biological reagents that produce biocompatible NPs using cost effective methods [7]. GNPs have been successfully synthesized from different biological sources such as proteins, flagella, bacteria and fungi [8,9,10,11]. Among these biological entities, plant extracts are extensively used in the synthesis of GNPs, because they are easier to handle, more readily available, cheaper and safer compared to the other aforementioned biological sources [7,12,13,14,15,16]. The synthesis of metal NPs using plant extracts is mediated through the presence of numerous reducing phytochemicals such as proteins, amines, phenols, carboxylic acids, ketones, aldehydes, etc. [17]. 

Several studies have reported the antimicrobial activities of biogenic GNPs. GNPs synthesized from natural honey exhibited significant antibacterial activity against pathogenic bacteria including multi-drug resistant bacterial strains [18]. Ayaz Ahmed et al. (2014) reported a potent antibacterial activity against several pathogenic bacteria such as *Pseudomonas aeruginosa* and *Escherichia coli* for the GNPs synthesized from the Indian plant, *Salicornia brachiata* [19]. *E. coli* and *Staphylococcus aureus* were also found to be sensitive to GNPs synthesized from *Mentha piperita* [20]. The synthesis of GNPs using extracts produced from plants with known antibacterial activities can potentially produce NPs with significant antibacterial activities.

*Galenia africana* L. var. *africana*, locally known as “kraalbos” or “geelbos”, is a common plant found throughout Namaqualand, South Africa [21]. This plant is used to treat venereal sores, asthma, coughs and eye infections. Indigenous tribes use the leaves from this plant to relieve toothache [22]. *Hypoxis hemerocallidea* is also an important medicinal plant that is indigenous to South Africa. Its corms are used in traditional medicine to treat psychiatric disturbances and as a diuretic. It is also used to kill small vermin and to treat gall sickness in cattle [23]. The infusion of this plant is widely exploited by the Zulu tribe to cure impotency [24]. Moreover, the extracts of *H. hemerocallidea* are used to treat many diseases including diabetes, urinary infections, cancer and in the management of Human Immunodeficiency Virus infection and Acquired Immune Deficiency Syndrome HIV/AIDS [25]. In addition to these medicinal uses, both *G. africana* and *H. hemerocallidea* plants are also known for their wound healing properties. A lotion from *G. africana* decoction is used to alleviate inflammation and to treat skin diseases [26]. *H. hemerocallidea* extracts can be applied topically to relieve skin wounds and rashes [27]. 

Microbial infections can deter the wound healing process as microbial pathogens can reduce the number of fibroblasts and collagen regeneration via activation of inflammatory mediators as a result of the production of microbial toxins [28]. Therefore, an ideal wound-healing agent should demonstrate antimicrobial activity. Both *G. africana* and *H. hemerocallidea* exhibited antibacterial activity, which could be potentially beneficial for wound healing. The 5,7,2-trihydroxyflavone isolated from *G. africana* has been found to have antibacterial activity against *Mycobacterium smegmatis* and *Mycobacterium tuberculosis* [29]. The acetone and the ethanolic extracts of *H. hemerocallidea* have been shown antibacterial activities against *S. aureus*. Also, different extracts of *H. hemerocallidea* exhibited efficient antibacterial activity against several bacterial strains. This activity was enhanced when the extracts of *H. hemerocallidea* were combined with other medicinal plant extracts [30]. All these studies demonstrate that the extracts of *G. africana* and *H. hemerocallidea* plants contain phytochemicals with antibacterial activity that could aid in the wound healing process.

In this paper, GNPs were synthesized from the aqueous extracts of *G. africana* and *H. hemerocallidea*. The synthesis of GNPs was monitored using Ultraviolet-Visible Spectroscopy (UV-Vis). The hydrodynamic size measurement of the GNPs was done using Dynamic Light Scattering (DLS). The GNPs’ morphology and their crystalline nature were inspected using High Resolution Transmission Electron Microscopy (HRTEM). Energy-Dispersive X-ray spectroscopy (EDX) was utilized to confirm the presence of the elemental gold in the GNPs. Additionally, the possible chemical functional groups involved in the biosynthesis of the GNPs were identified using Fourier Transform Infrared spectroscopy (FTIR). Thermogravimetric analysis (TGA) was also done to get an estimation of the amount of organic layer that surrounds the GNPs. The growth kinetics of the GNPs was also studied. The stability of the GNPs was measured in different biological buffer solutions. The in vitro toxicity of the synthesized GNPs was evaluated on non-cancerous human fibroblast cell line (KMST-6). The antibacterial evaluation of the GNPs and the extracts against several gram-positive and gram-negative bacteria was performed.

## 2. Results and Discussion

In order to produce GNPs chemically, a reducing agent is normally added to the gold salt to reduce gold atoms and allowing them to grow into GNPs. The addition of other organic molecules can be done to surround the GNPs in order to control their growth, prevent their aggregation and increase their stability [31]. 

The ability of *G. africana* and *H. hemerocallidea* plant extracts to provide secondary metabolites, not only capable of reducing the gold salt but also able to provide stabilization (capping) properties, was examined. The shape, distribution, morphology and surface charges of the GNPs were studied. The study also evaluated the biocompatibility and antibacterial activity of the GNPs. 

This study follows on from a previous report in which extracts from several indigenous South African plants were screened for the biosynthesis of GNPs using a quick and easy microtitre plate method [32]. The previously reported methodology was applied here in order to obtain the optimum concentration for each plant extract (as mentioned in Section 3) that can produce the smallest and most defined GNPs. In the previous report, it was also concluded that the use of high temperature facilitates the synthesis of smaller GNPs. Hence, the synthesis of the GNPs in the current study was done at 70 °C. 

### 2.1. UV-Vis Analysis

**Figure 1:**
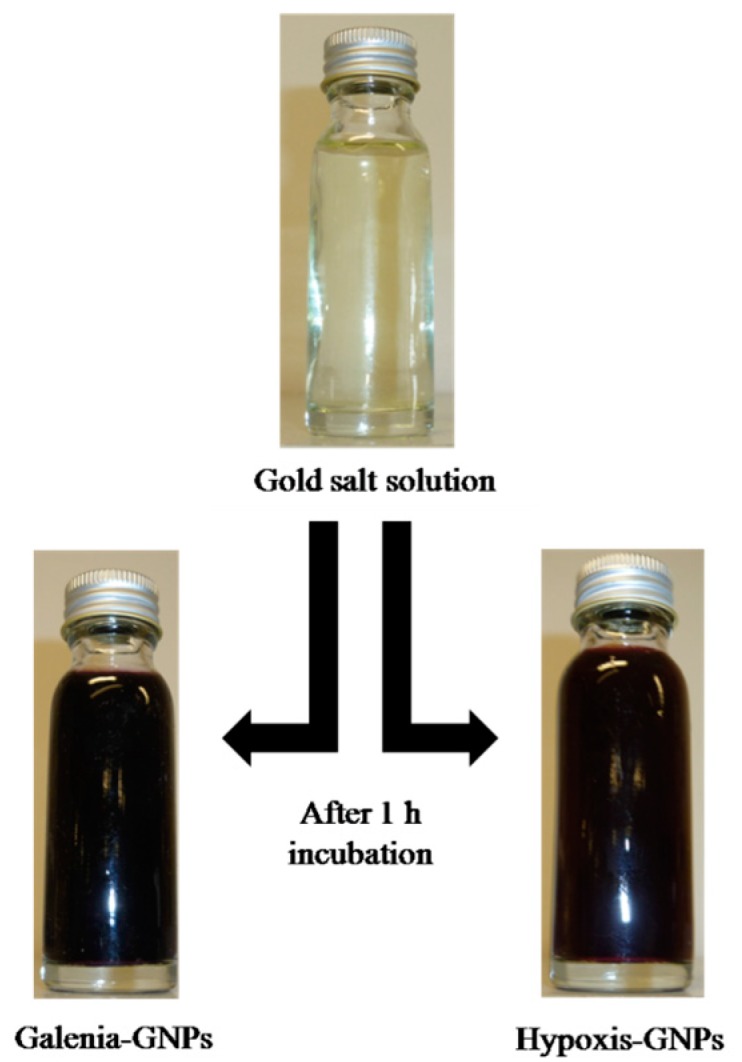
Digital photographs of the aqueous solutions of gold salt before the addition of the extracts, and Galenia-GNPs and Hypoxis-GNPs after 1 h incubation of the gold salt with the respective extracts.

**Figure 2:**
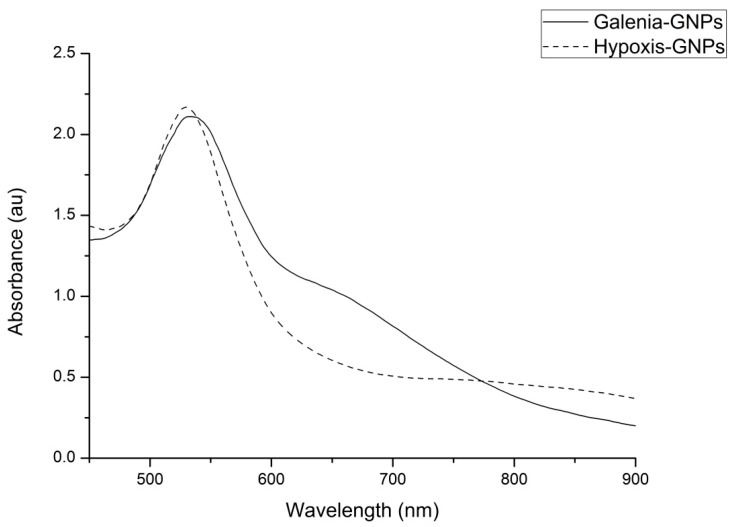
UV-Vis spectra of GNPs synthesized from *G. africana* (Galenia-GNPs) and *H. hemerocallidea* (Hypoxis-GNPs) plant extracts.

The visual observation of the color change from light yellow to red for the gold salt/plant extract mixtures after the 1 h incubation (Figure 1) is an indication that GNPs were formed. This confirmed that the extracts were able to reduce the Au^+3^ ions to Au^0^ by the secondary metabolites/phytochemicals present in the extracts [15]. The cause of this red color in the GNPs’ colloidal solution, which is not observable in the bulk material or the individual atoms, is a result of the oscillation of free conduction electrons known as Surface Plasmon Resonance (SPR) [33]. A UV-Vis spectrum with a maxima absorbance between 500 and 600 nm is indicative of GNPs formation [34]. Figure 2 shows the UV-Vis spectra of GNPs from Galenia-GNPs (GNPs produced from *G. africana*) and Hypoxis-GNPs (GNPs produced from *H. hemerocallidea*). Galenia-GNPs and Hypoxis-GNPs exhibited a maximum absorbance (λ_max_) of 534 ± 2 nm and 530 ± 1 nm, respectively. Several factors such as the size and the shape of the NPs, the refractive index of the medium and the inter-particle distances affect the shape and position of the GNPs’ SPR in the UV-Vis spectrum [35]. In Figure 2, the band generated by Hypoxis-GNPs was sharper and more symmetrical with small absorption after 600 nm as opposed to Galenia-GNPs’ band, which can be a sign of better uniformity in size distribution of Hypoxis-GNPs compared to Galenia-GNPs [36]. Further, the absorption tail in the Near Infrared (NIR) wavelength observed for Galenia-GNPs could be caused by the excitation of the in-plane SPR and can be a result of anisotropic GNPs [37] or the deviation from spherical geometry of the GNPs [38]. These results may indicate the presence of more effective capping agents in *H. hemerocallidea* plant extract compared to *G. africana* that prevented the aggregation of the GNPs and enhanced their uniformity. However, the GNPs with absorption in the NIR region have been found to be useful in several biomedical applications and in the fabrication of photonic devices such as optical sensors [39].

**Figure 3:**
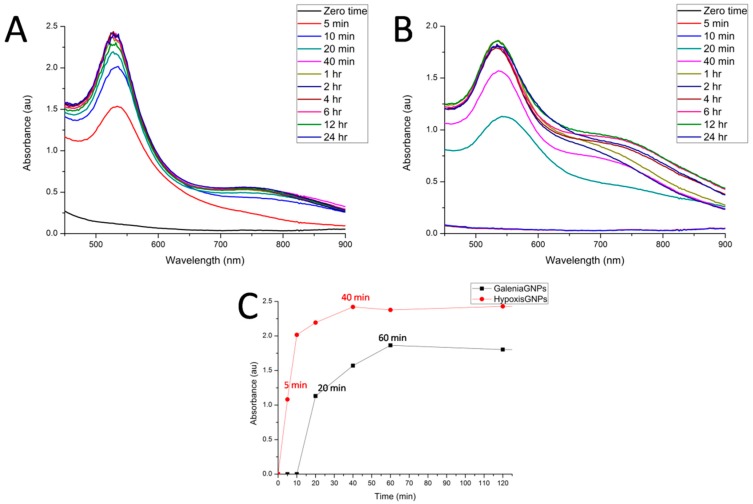
The UV-Vis spectra recorded as a function of time of GNPs synthesized from (**A**) *H. hemerocallidea* and (**B**) *G. africana*; (**C**) shows the λ_max_ values of the two GNPs as a function of time.

We also studied the kinetics of GNPs formation by examining the changes in the λ_max_ of the plant extract/gold salt mixtures over time. Hypoxis-GNPs started to form and show λ_max_ above 1 Absorbance unit (au) after 5 min (Figure 3A). This increase in λ_max_ is the result of the increasing number of GNPs as Au^+3^ ions are reduced to Au^0^ [40]. The Hypoxis-GNPs reached a maximum value after 40 min and thereafter remained unchanged suggesting the reaction was complete at 40 min (Figure 3C). On the other hand, the reaction with the *G. africana* plant extract started to change color and the λ_max_ increased above 1 au only after 20 min (Figure 3B) indicating the presence of lower reduction power phytochemicals in *G. africana* extract compared to *H. hemerocallidea*’s. Both GNPs exhibited constant λ_max_ from 60 min (Figure 3C), which show that 1 h of incubation was sufficient to complete the reaction for both plant extracts.

### 2.2. Particle Diameter and Particle Size Distribution Analysis

**Figure 4:**
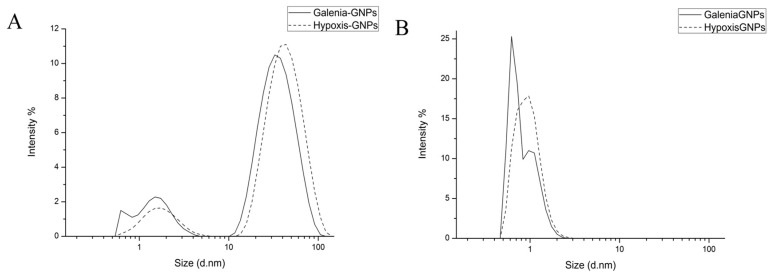
DLS distribution curves of GNPs’ hydrodnamic diameter by (**A**) intensity and (**B**) number of GNPs.

The distribution of the hydrodynamic diameters of the GNPs was measured by two different DLS-based techniques (based on size by intensity and by the number of GNPs) using the Zetasizer (Malvern Instruments Ltd., Malvern, UK). The size distribution based on intensity is depicted in Figure 4A, in which the scattering intensity is plotted against the logarithms of the particle diameter. Hypoxis-GNPs showed bimodal distribution, whereas Galenia-GNPs showed multimodal distribution that indicates the anisometric nature of Galenia-GNPs compared to Hypoxis-GNPs (Figure 4A). This may also explain the presence of NIR absorption peak in the UV-Vis spectrum of the Galenia-GNPs (Figure 2). In both GNPs, the peak intensity of the large particles was higher than the peak intensity of the small particles, which was expected since the particle size distribution based on the light-scattering intensity is greatly influenced by larger particles [41]. Conversely, the peaks for small particles showed higher intensity in the number-weight based size distribution (Figure 4B), with no intensity observed for larger particles. It should be taken into consideration that the error in the data obtained from number-weight size distribution is large due to its sixth power dependence on the original scattering intensity data. Yet, it can be a useful tool to compare the distribution of the two plant extracts’ GNPs of small size. From Figure 4B it can be observed that the two plant extracts were able to synthesize very small GNPs (1–2 nm) in which a higher number of small size GNPs could be synthesized with *G. africana* extract as opposed to *H. hemerocallidea* as can be deduced from the intensity of the peaks.

Table 1 shows the average diameters of light-scattering intensity peaks shown in Figure 4A as well as the Z-average diameter, which is derived from the light-scattering intensity data. In agreement to the distribution curves, the Z-average diameter of the Galenia-GNPs have smaller average diameter in comparison to Hypoxis-GNPs.

### 2.3. FTIR Analysis

The FTIR analysis was done for the plant extracts and the GNPs to identify the possible functional groups involved in the biosynthesis of GNPs. This information can aid in identifying the phytochemicals involved in the reduction of the gold salt and may also provide useful information on how to conjugate other chemical entities (e.g., small molecule drugs, peptides, nucleic acids, etc.) onto the GNPs for biomedical applications. The bio-reduction mechanism of gold ions using plants extracts continues to be elucidated, despite the increasing attention being given to the biogenic synthesis of the GNPs [42]. Several studies suggest that various phytochemicals may play a role in the synthesis of GNPs [42,43]. Generally, different chemical classes were found to influence the production of the GNPs based on the major constituent of each plant extract [32].

**Figure 5:**
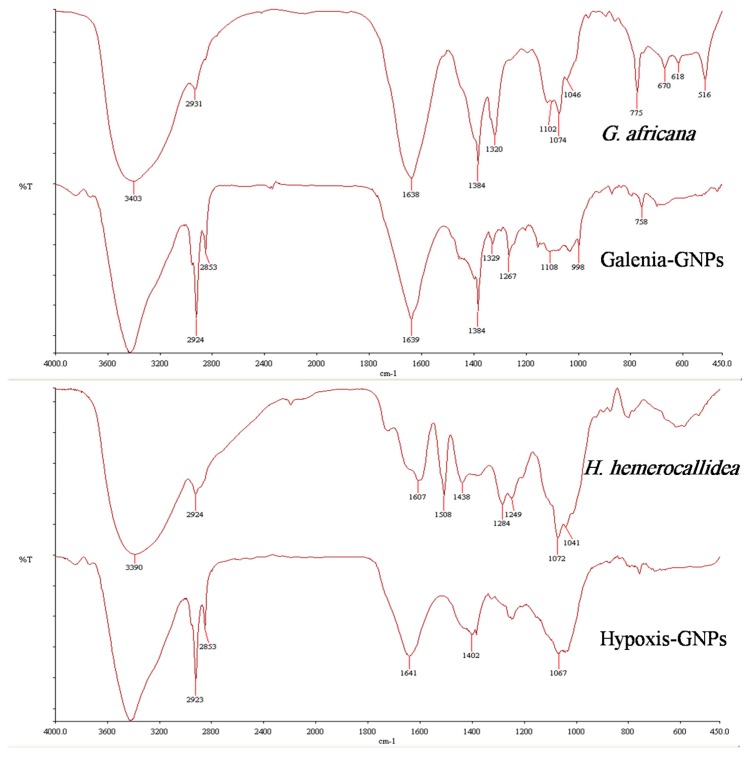
FTIR spectra of *H. hemerocallidea* and *G. africana* and their respective GNPs.

Figure 5 shows the FTIR spectra of both the plant extracts and the GNPs. Both GNPs showed similarities with their respective extracts, which may be due to the presence of similar compounds in both the extracts and the GNPs. Additionally, some bands of the FTIR spectra of the GNPs appeared to be shifted when compared to the FTIR spectra of the extracts. These shifts were expected and are believed to be caused by the influence of the nearby metal and possibly suggest the involvement of the corresponding functional groups in the GNPs synthesis [41]. These observed shifts are highlighted in Table 2, which also shows the possible functional groups involved in the synthesis of the GNPs from both extracts. Interestingly, some major peaks were generated in the FTIR spectra of both GNPs indicating that similar functional groups are key players in the synthesis of the GNPs. For instance, the FTIR spectra of Galenia-GNPs and Hypoxis-GNPs revealed similar broad bands at 3428 and 3420, respectively, which represents the O–H group of alcohols [12]. The intense band at ~ 2924 cm^−1^ can be a result of asymmetric stretching of the C–H group [12]. Also, the peak centered at 1384 cm^−1^, which indicates the presence of the –CH_3_ group of alkanes, was also recorded in Galenia-GNPs. Galenia-GNPs also demonstrated a peak at 1329 cm^−1^ that corresponds to an alcoholic or phenolic O–H group [41]. The transmittance of O–H and C–O bands in the FTIR spectra indicates the presence of hydroxyl and carbonyl groups on the GNPs possibly as a result of the involvement of flavonoids, terpenoids, phenolic compounds and/or carbohydrates in the GNPs biosynthesis (Table 2). Several studies reported the role of these hydroxyl and carbonyl containing compounds in the reduction, capping and stabilization of the GNPs [12,44]. Amino acids and proteins were also suggested to act as stabilizers of GNPs after the reduction step [13]. Yet, a quick phytochemical screening, using the Biuret and Ninhydrin tests, showed that both aqueous extracts were negative for the presence of proteins and amino acids, and hence we postulate that proteins and amino acids do not play a role in the stabilization of the GNPs in this study. 

The chemical study of *G. africana* revealed that this plant is rich in flavonoids [21,22]. Indeed, the FTIR spectrum of *G. africana* aqueous extract showed a strong band at 1384 cm^−1^ that corresponds to the phenolic O–H group and hence we speculate that the flavonoids of this plant are responsible for the reduction of the gold salt to produce Galenia-GNPs. Further, *H. hemerocallidea* is well known for producing a variety of hydroxyl-rich phytoglycosides [27]. A study by Jung et al. (2014) reported the synthesis of GNPs from several glycosides and concluded that the GNPs can be reduced as a result of the oxidation of C-6-OH in the sugar unit into carboxylic acid [45]. The presence of the shifted band at 1267 cm^−1^, in the FTIR spectrum of Hypoxis-GNPs, which can be attributed to the C–O group of carboxylic acids, may be a result of the oxidation of the aforementioned oxidation site (Table 2). One of the major secondary metabolites of *H. hemerocallidea* is Hypoxoside, which is a phytoglycoside compound containing the same oxidation site reported by Jung et al. (2014). Hence, we also speculate that Hypoxoside may play a major role in the synthesis of the GNPs. Clearly, these major compounds should be isolated and tested for the synthesis of the GNPs in order to identify, with certainty, the actual functional groups responsible for the synthesis of the GNPs from each plant. This investigation is ongoing.

### 2.4. HRTEM and EDX Analysis

**Figure 6:**
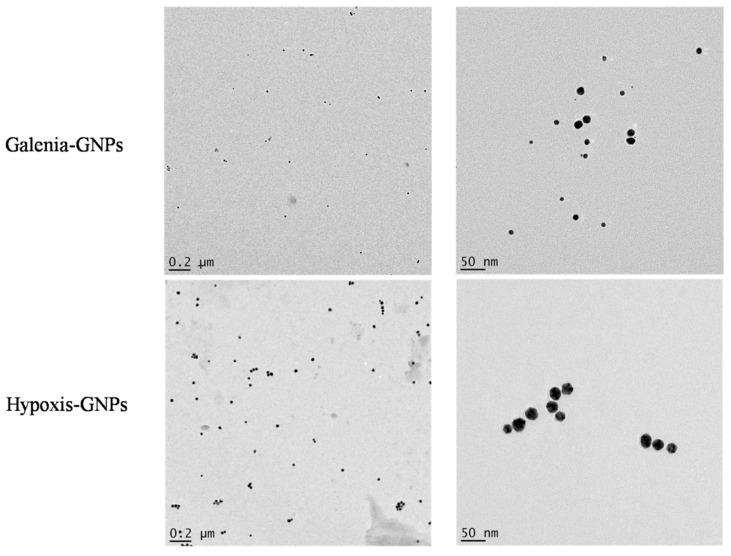
HRTEM images of Galenia-GNPs and Hypoxis-GNPs.

**Figure 7:**
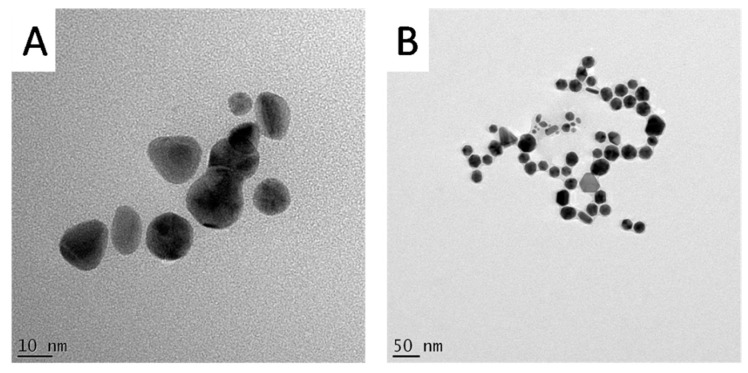
HRTEM images showing spherical deviations observed in (**A**) Galenia-GNPs and (**B**) Hypoxis-GNPs.

The HRTEM analysis of the GNPs was done to study their morphologies, crystalline nature and their particle size distribution. Interestingly, the HRTEM images show predominance of spherical GNPs from the two plant extracts (Figure 6). Due to the presence of numerous phytochemicals in the extracts that are capable of reducing the gold salt, it is common that plant phytochemicals produce GNPs with a mixture of geometrical shapes [32]. It is suggested that the presence of strong interaction forces between the capping bio-molecules and the surfaces of GNPs could keep the nascent GNPs from sintering, resulting in small sized spherical GNPs [33]. Therefore, the synthesis of spherical shapes in this study may imply that the capping agents, present in *H. hemerocallidea* and *G. africana*, exhibit strong interaction with the newly grown GNPs and prevent them from developing into other shapes. Yet, some deviations from the spherical shapes were observed in the HRTEM images of the two GNPs (Figure 7). These deviations, which were more common in Galenia-GNPs, may explain the absorbance peak beyond 600 nm in the UV-Vis spectra of Galenia-GNPs in Figure 2.

**Figure 8:**
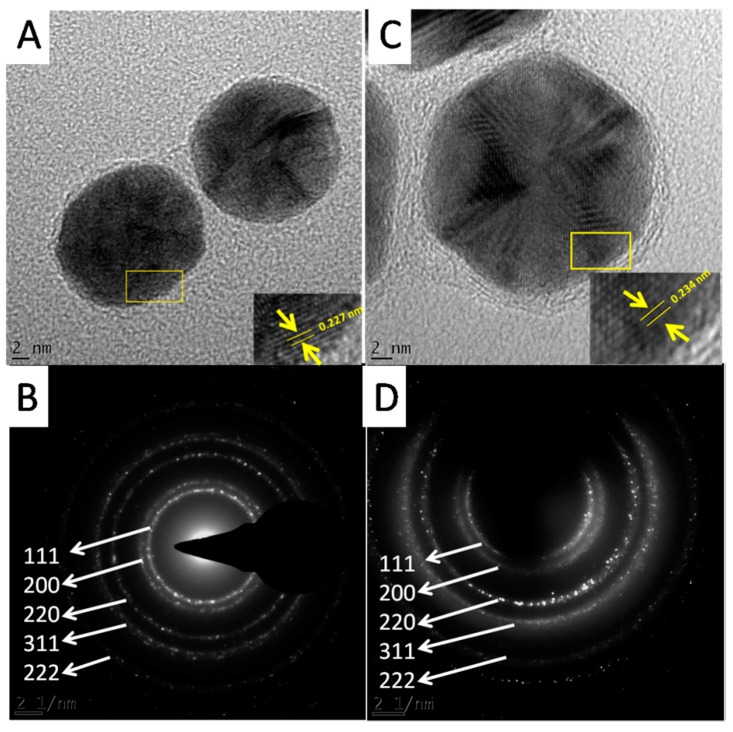
HRTEM images showing fringe lattics observed in (**A**) Galenia-GNPs and (**C**) Hypoxis-GNPs, and SAED pattern of (**B**) Galenia-GNPs and (**D**) Hypoxis-GNPs.

The HRTEM analysis also revealed the crystalline nature of the GNPs. Figure 8A,C show the lattice fringes of the two GNPs. The shortest lattice distances were 0.234 and 0.227 nm for Hypoxis-GNPs and Galenia-GNPs, respectively (Figure 8A,C). These values correspond approximately to the interplanar spacing between (111) planes of gold [46]. The crystalline nature of the GNPs was also confirmed by the selected area electron diffraction (SAED). The bright rings were found to correspond to the (111), (200), (220), (311) and (222) planes of the gold (Figure 8B,D). 

**Figure 9:**
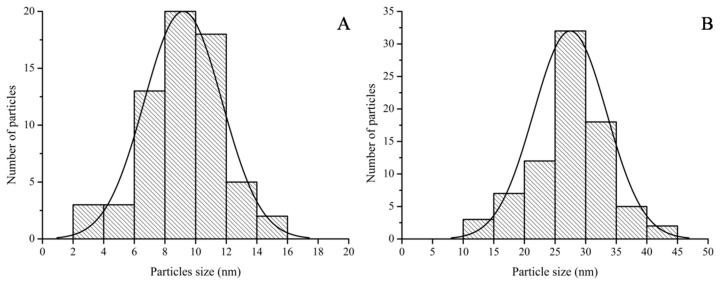
Particle size distributions of (**A**) Galenia-GNPs and (**B**) Hypoxis-GNPs as obtained from the HRTEM images.

The particle size distributions obtained from the HRTEM images were similar to the DLS data (Figure 9). The particle size range of Galenia-GNPs was between 2 and 16 nm with the largest number of the particles being between 8 and 10 nm in diameter (Figure 9A). On the other hand, the particle size of Hypoxis-GNPs ranged from 10 to 45 nm with the majority of the NPs being between 25 and 30 nm in diameter (Figure 9B). Also, the average particle size of Galenia-GNPs (9 ± 2 nm) was smaller than those of Hypoxis-GNPs (27 ± 6 nm) as obtained by HRTEM analysis. It must be noted that the hydrodynamic particle size data obtained from the DLS analysis is usually larger than the particle size determined by HRTEM [12]. Indeed, the average size of Galenia-GNPs obtained by HRTEM was smaller than the average size determined by Zetasizer. Conversely, the average size of Hypoxis-GNPs as determined by HRTEM was slightly larger than the size obtained using the Zetasizer. Yet, it must be taken into consideration that only a few NPs are shown in each frame of the HRTEM images, so any shape and size distributions determinations of the GNPs using HRTEM images will not be completely statistically reliable [41].

The concentration of GNPs in this study was determined using their UV-Vis data as reported [47]. Using multipole scattering theory, Haiss and co-workers (2007) showed that the optical properties of the spherical GNPs are dependent on the particle size. As a result, the authors showed that the molar concentration and particle size of the GNPs could be deduced from their UV-Vis spectra. Hence, it was useful to measure the difference in the particle size data obtained using the three methods applied in this study, i.e., Zetasizer, HRTEM and UV-Vis spectra (Table 3). The results in Table 3 confirmed that three techniques showed that Galenia-GNPs were smaller in size when compared to Hypoxis-GNPs. The difference in size between Galenia-GNPs and Hypoxis-GNPs as determined by the Zetasizer and HRTEM was 15 nm and 18 nm, respectively, while the size determined using the UV-Vis spectra was 8 nm.

**Figure 10:**
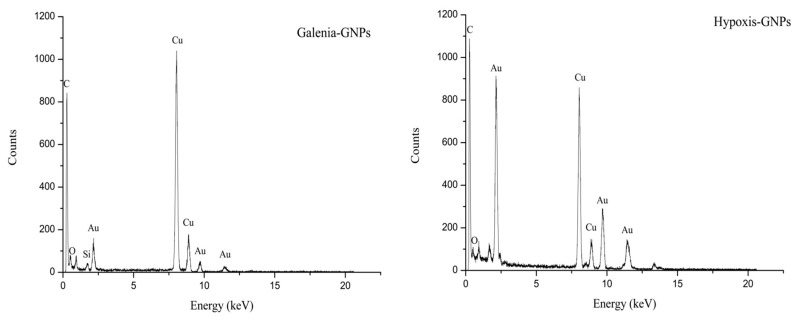
EDX spectra of Galenia-GNPs and Hypoxis-GNPs.

The presence of the elemental gold was confirmed in the graphs obtained from the EDX spectroscopy analysis of the GNPs. The EDX data showed adsorption of gold peaks at around 2.3, 9.7 and 11.3 keV (Figure 10). These values are in agreement with a previous study [48]. The presence of carbon, copper and silicon peaks in the samples is attributed to the HRTEM grid and/or the detector window [49]. On the other hand, traces of the phytochemicals of the extracts present around the GNPs or in the medium may have caused the presence of oxygen peaks [13].

### 2.5. Thermal Study

**Figure 11:**
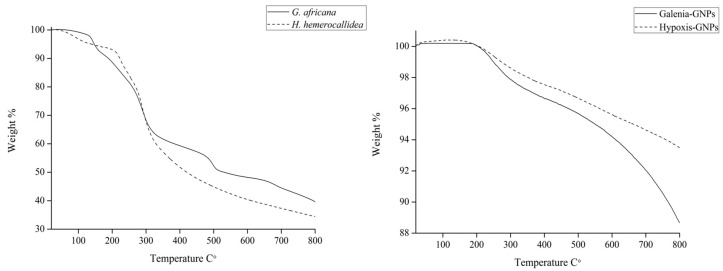
TGA data for *H. hemerocallidea* and *G. africana* and their respective GNPs.

The TGA was done in order to determine the percentage of the organic matter (phytochemicals involved in the synthesis) present in the GNPs. The weight loss of 5 mg of the GNPs and the extracts was measured between 20 and 800 °C (Figure 11). Table 4 summarizes the weight loss percentage of the extracts and the GNPs at different temperatures. Unlike the extracts, both GNPs did not show weight loss at 100 °C. Any weight loss at this temperature is believed to be a result of the loss of evaporation of adsorbed water [50]. It is expected that most organic compounds and functional groups will be completely burned off at 400 °C [51,52]. Table 4 shows that Hypoxis-GNPs and Galenia-GNPs, respectively, only lost 2.5% and 3.4% of their weight at 400 °C. Both of the extracts showed nearly 50% weight loss at the same temperature. The thermal decomposition of resistant aromatic compounds and biogenic salts is expected to occur at temperatures beyond 400 °C [50]. Thus, the lower weight loss in the case of *G. africana* may be as a result of the presence of higher content of these heat resistant compounds. At 800 °C Galenia-GNPs showed more weight loss (4%) when compared to Hypoxis-GNPs. It should be noted that the amount of the *G. africana* extract used in the synthesis of Galenia-GNPs was twice the amount of *H. hemerocallidea* extract used to synthesize the Hypoxis-GNPs (as mentioned in Section 3) and it was therefore expected that the weight loss value of Galenia-GNPs would be higher compared to Hypoxis-GNPs. 

### 2.6. Stability of the GNPs

To understand the stability of the GNPs, the zeta potential values, measured immediately after the synthesis, were obtained using the Zetasizer. Hypoxis-GNPs and Galenia-GNPs demonstrated negative zeta potential values of −22 and −20, respectively. These negative values can estimate the long-term stability of the GNPs in a solution, as they can provide enough repulsion forces between the particles and prevent their agglomeration [53].

**Figure 12:**
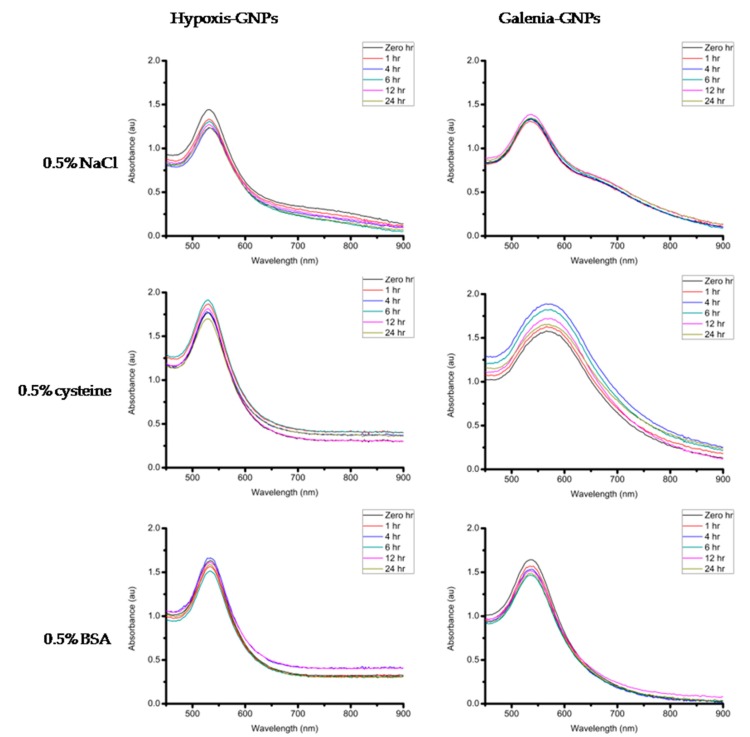

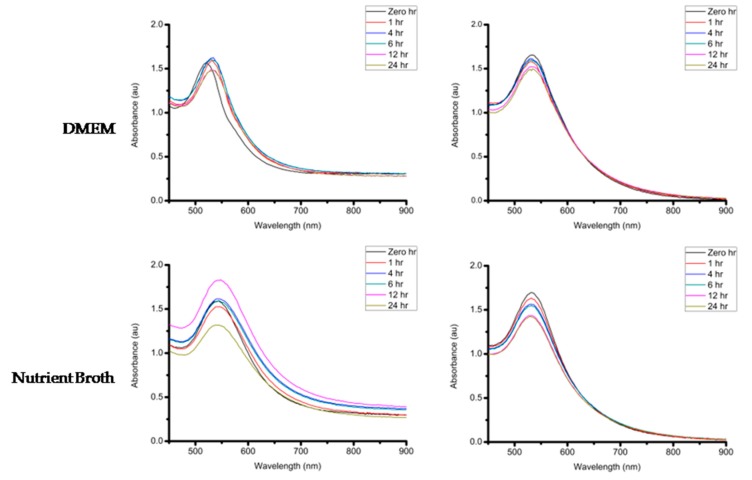
UV-Vis spectra of the GNPs taken over a 24-h period in buffers containing 0.5% NaCl, 0.5% cysteine and 0.5% BSA, Nutrient broth and in DMEM supplemented with 10% FBS.

If these GNPs are to be considered for biomedical applications, they must maintain their stability in different buffer solutions (e.g., Sodium Chloride (NaCl), cysteine and Bovine Serum Albumin (BSA)). The stability of the GNPs was measured after incubation with the aforementioned buffer solutions as well as the growth media used in the biological assays in this study (Dulbecco’s Modified Eagle’s Medium (DMEM) supplemented with 10% Fetal Bovine Serum (FBS) and Nutrient broth). The GNPs were incubated at 37 °C with DMEM and Nutrient broth in order to determine the effect of the media on the stability of the GNPs under experimental conditions. A minimal change in the UV-Vis spectra of the GNPs is an indication of the GNPs stability. When the GNPs lose stability they may precipitate, which can be observed by the significant red shifts and broadening of the UV-Vis bands [54]. After measuring the UV-Vis of the two GNPs incubated with different buffers and the biological media over a 24 h period, it was observed that these GNPs were generally stable in most of the buffer conditions tested with no changes in the UV-Vis bands (Figure 12). One exception was the effect of 0.5% cysteine on Galenia-GNPs, which caused the UV-Vis bands to become broader at all the time-points. Nonetheless, Galenia-GNPs showed excellent stability in DMEM that usually contains cysteine and other amino acids but at lower concentrations.

### 2.7. Antibacterial Effects and Toxicity of the GNPs

Generally, the antibacterial effect of different plant extracts is well documented and recognized. However, the antibacterial effect of the biogenic metal NPs produced from such plant extracts still remains largely unexplored and can prove useful in the search for new antibacterial agents [12]. For this reason, the antibacterial activities of Hypoxis-GNPs, Galenia-GNPs along with the tested plant extracts were investigated. The Alamar blue assay was used to measure the bacterial growth after treatment. Resazurin (Alamar blue dye) undergoes colorimetric change in response to cellular metabolic reduction to give the highly fluorescent compound resorufin that can be quantified by measuring its fluorescence [55]. The Minimum Inhibitory Concentration (MIC) in this study was defined as the lowest concentration of the tested samples that significantly (*p* < 0.05) inhibits the growth of the tested bacterial strains as compared to the negative control value. The antibacterial evaluation was done against a panel of gram-positive and gram-negative bacterial strains that are known to cause wound infections.

The MIC values for the GNPs and the plant extracts are summarized in Table 5. The antibiotic Ampicillin was included as a positive control. The antibacterial effect of citrate-capped GNPs was also tested. Hypoxis-GNPs demonstrated significant antibacterial activity against the tested bacterial strains when compared to Galenia-GNPs. Interestingly, the MIC value of the Hypoxis-GNPs (32 nM) was the same for the bacterial strains. However, the viability of the bacteria at this MIC value varied between the different bacterial strains. For instance, *P. aeruginosa* was the most susceptible by Hypoxis-GNPs and showed the lowest viability with 10 ± 1% compared to 16 ± 1%, 20 ± 1% and 43 ± 5% for *E. coli*, *Staphylococcus epidermidis* and *S. aureus*, respectively. Galenia-GNPs only showed an antibacterial effect on *P. aeruginosa* with a MIC value of 32 nM and viability of 35 ± 5%. Further, none of the aqueous plant extracts induced any growth inhibition in this study. However, Ncube et al. (2012) reported that the *H. hemerocallidea* aqueous extract have an MIC value of 12.5 mg/mL against *S. aureus* and *E. coli*, which is significantly higher than the highest concentration tested in this study [30]. Katerere and Eloff (2008) also reported that the acetone corm extract of this plant had a low MIC value of 0.31 mg/mL against *S. aureus* [56]. This difference is likely to be attributed to the difference in the chemical nature of phytochemicals present in the acetone and water extracts. The citrate-capped GNPs failed to induce similar antibacterial activity as observed with the biogenic GNPs. Also, the MIC values obtained for Ampicillin were within the ranges reported in previous studies [57,58,59]. The MIC for Ampicillin was significantly higher for *P. aeruginosa* (2 mg/mL) compared to the other bacterial strains tested. The increased resistance of *P. aeruginosa* to Ampicillin is possibly due to changes in the penicillin-binding proteins, membrane impermeability and the production of beta-lactamases [60].

It is thought that the smaller size of the GNPs compared to the size of the bacterium enables the GNPs to exert bacterium cell death by adhering to its cell wall [61]. The GNPs can then penetrate the cell wall of the bacterium and induce death by affecting respiratory mechanisms and cell division by binding to protein- or phosphorus-containing compounds, such as DNA [62]. It is believed that the variation in activity against the bacterial strains is dictated by the nature of the bacterial cell wall. The cell wall of the gram-positive bacterial strains, for example, has a thicker peptidoglycan layer compared to the cell wall of the gram-negative bacteria [63]. As a result, GNPs can penetrate the cell wall of the gram-negative bacteria and exert their antibacterial action more easily than in gram-positive bacteria [64]. Accordingly, the variation in the viability of the bacterial strains after treatment with the MIC value of Hypoxis-GNPs could be attributed to the nature of the cell wall composition. In fact, growth inhibition caused by the Hypoxis-GNPs was more significant in the two gram-negative bacterial strains (*E. coli* and *P. aeruginosa*). 

Furthermore, the results show that the aqueous plant extracts lacked any bacterial activity at the highest concentration tested in this study in contrast to the biogenic GNPs. It is, however, possible that a higher concentration of the extracts can be active against these bacterial strains as demonstrated previously [30]. The lower antibacterial activity of the extracts compared to the GNPs may be due to the fact that either the bacteria are adopting resistance mechanisms against the free phytochemicals or there is some synergistic activity between the GNPs and the capping phytochemicals [65]. The higher antibacterial activity of the biogenic GNPs may also be associated with the increase in the concentration of the active phytochemicals capping the GNPs. Consequently, when bacteria are exposed to the GNPs an augmented antibacterial effect is obtained. It is also possible that the GNPs have more targeting effect or higher affinity towards the bacterial cells in comparison to the free phytochemicals. The fact that the non-phytochemical capped GNPs (Citrate NPs) was not as active as Hypoxis- or Galenia-GNPs further supports the role of the phytochemicals in inhibiting the bacterial growth. In addition, it is known that the antibacterial activity of the NPs is inversely proportional on their particle size [66], yet the bigger size Hypoxis-GNPs were more active against the bacteria than the smaller particle size Galenia-GNPs. Hence, it could be speculated that the properties of the phytochemicals capping the NPs are an important factor in determining the antibacterial activity regardless of the size of the GNPs. 

**Figure 13:**
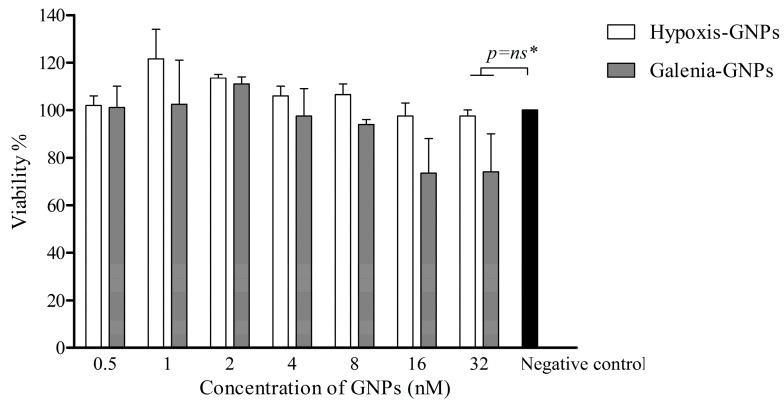
The effect of the GNPs on the cell viability of KMST-6 as determined by the MTT assay. * No statistically significant difference (*p* > 0.05) compared to the negative control.

In view of the fact that biogenic GNPs such as Hypoxis-GNPs and Galenia-GNPs can potentially be applied in wound dressings to protect the exposed tissue against bacterial infections, we also investigated the potential toxicity of these GNPs to the human fibroblast cells. The toxicity of the GNPs towards the KMST-6 cell line therefore was established using in vitro cell culture testing. Figure 13 shows that there was no significant reduction in the viability of KMST-6 cells after a 24 h treatment with different concentrations (up to 32 nM) of the GNPs, which is equivalent to the MIC values obtained for the GNPs against some of the bacterial strains. This preliminary data suggests that these GNPs are safe for therapeutic use.

## 3. Materials and Methods

### 3.1. Materials

The aqueous extract of *H. hemerocallidea* was purchased from Afriplex (Cape Town, South Africa). Polystyrene 96-well microtitre plates were obtained from Greiner bio-one GmbH (Frickenhausen, Germany). Ampicillin, 3-[4,5-dimethylthiazol-2-yl]-2,5-diphenyl tetrazolium bromide (MTT) and gold salt (sodium tetrachloroaurate (III) dihydrate) were purchased from Sigma-Aldrich (Cape Town, South Africa). *N*-Acetyl-L-cystein and Alanin were purchased from Boehringer Mannheim GmbH (Mannheim, Germany). DMEM, penstrep (penicillin–streptomycin) and Phosphate buffered saline (PBS) were purchased from Lonza (Cape Town, South Africa). BSA was procured from Miles Laboratories (Pittsburgh, PA, USA). FBS was bought from Thermo Scientific (Ansfrere, South Africa). Nutrient broth and Miller Hinton agar were purchased from Biolab (supplied by Merck, Modderfontein, South Africa). Alamar blue dye was obtained from Invitrogen Corporation (San Diego, CA, USA). NaCl, Sodium Hydroxide (NaOH) and Ninhydrin reagent were brought from Merck (Cape Town, South Africa). Citrate-capped GNPs (14 nm) were obtained from DST/Mintek Nanotechnology Innovation Centre (Gauteng, South Africa).

### 3.2. Preparation of G. africana Aqueous Extract

*G. africana* was collected during the month of May 2015 from the Western Cape Province, South Africa. The plant was identified by Dr. Chris N. Cupido, the co-author of this paper, and a specimen was deposited in Kirstenbosch National Botanical Garden (Cape Town, South Africa) under accession number 1468255/NBG. The fresh aerial parts of *G. africana* were dried in the shade. To obtain the aqueous extract, 50.0 mL of boiled distilled water were added to 5.0 g of the dried plant powder. Afterwards, the plant decoction was centrifuged for 2 h at 3750 rpm using an Allegra^®^ X-12R centrifuge (Beckman Coulter, Cape Town, South Africa). The supernatant was then filtered through 0.45 μm filters and freeze-dried using FreeZone 2.5 L freeze-dryer (Labconco, Kansas City, MO, USA). 

### 3.3. Biogenic Synthesis of the GNPs and Their Characterization

The *H. hemerocallidea* and *G. africana* plant extracts were first screened for the production of GNPs in 96-well microtitre plates using the method reported in a previous study [32]. In short, 250 μL of 1.0 mM sodium tetrachloroaurate (III) dihydrate were mixed with 50.0 μL of each plant extract in a 96-well microtitre plate (the concentrations of the extracts varied from 8.0 to 0.125 mg/300 μL). The plate was incubated for 1 h at 70 °C with shaking at 40.0 rpm. The production of the GNPs was monitored by measuring the UV-Vis spectra (450–900 nm) using a POLARstar Omega microtitre plate reader (BMG Labtech, Cape Town, South Africa). For further evaluations of the GNPs, the volume of the gold salt and plant extracts mixtures was up-scaled after determining the optimum concentrations of the plant extracts that produce desirable GNPs (0.5 mg/300 μL for *G. africana* and 0.25 mg/300 μL for *H. hemerocallidea*). The GNPs were then centrifuged and the pellets were washed trice with distilled water and ultimately re-suspended in distilled water.

### 3.4. DLS Analysis

The zeta potential and hydrodynamic size values of the freshly synthesized GNPs were measured using a Zetasizer (Malvern Instruments Ltd., Malvern, UK) at 25 °C and a 90° angle. Zetasizer software version 7.11 was used to analyze the data. 

### 3.5. FTIR Spectroscopy

The FTIR analysis was done using PerkinElmer spectrum one FTIR spectrophotometer (Waltham, MA, USA) according to the method reported previously [7]. The freeze-dried GNPs and the extracts were added to KBr powder and pressed into a round disk. A pure KBr round disk was used for background correction.

### 3.6. HRTEM and EDX Analysis

One drop of the GNPs solution was added onto a carbon coated copper grid. The grids were allowed to dry for a few minutes under a Xenon lamp. The HRTEM images were obtained using FEI Tecnai G^2^ 20 field-emission gun (FEG) HRTEM operated in bright field mode at an accelerating voltage of 200 kV. The elemental composition of the GNPs was identified using EDX liquid nitrogen cooled Lithium doped Silicon detector.

### 3.7. Image Processing

The image analysis software ImageJ 1.50b version 1.8.0_60 (http://imagej.nih.gov/ij) was used to analyze the HRTEM images.

### 3.8. TGA

The TGA was done using PerkinElmer TGA 4000 (Waltham, MA, USA). The freeze-dried GNPs (5.0 mg) or plant extracts (5.0 mg) were heated from 20 to 800 °C in nitrogen atmosphere (flow rate was 20.0 mL/min) [67]. The temperature was increased at a rate of 10 °C/min.

### 3.9. Stability Evaluation of the GNPs

To measure the effect of different aqueous buffer solutions (e.g., 0.5% NaCl, 0.5% cysteine and 0.5% BSA) on the stability of the biogenic GNPs, 100 µL of the GNPs solutions were mixed with 100 µL of the buffer solutions in a 96-well microtitre plate. The stability of the GNPs was also evaluated in DMEM (supplemented with 10% FBS) and Nutrient broth. The stability of the GNPs was monitored by measuring the UV-Vis spectrum (between 450 and 900 nm) of the samples at 1, 4, 6, 12 and 24 h after mixing the GNPs with the buffer solutions or the media.

### 3.10. Phytochemical Screening

#### 3.10.1. Test for Proteins (Biuret Test)

The phytochemical assays were done as described previously with minor modifications [68]. To test the aqueous extracts for the presence of proteins, a few drops of 5.0% NaOH and a few drops of 1.0% Cu(SO_4_)_2_ were added to 2.0 mL of each aqueous extract. BSA was used as a positive control. A violet color change indicated the presence of proteins.

#### 3.10.2. Test for Amino Acids (Ninhydrin Test)

Few drops of Ninhydrin reagent were added to 2.0 mL of the aqueous extracts. The mixtures were heated in water bath for 10 min. Alanin was used as a positive control. The formation of purple color indicated the presence of amino acids. 

### 3.11. Cytotoxicity Evaluation of the GNPs

The toxicity of the GNPs was tested on the non-cancerous human fibroblast cell line (KMST-6). The cells were maintained in DMEM containing 10% FBS and 1% penstrep in a 37 °C humidified incubator with 5% CO_2_ saturation. The viability of the KMST-6 cells was evaluated using the MTT assay as described by Mmola and co-workers with some modifications [65]. The cells were seeded in a 96-well microtitre plates at a density of 2.0 × 10^4^ cells/100 μL/well. The plates were incubated at 37 °C in a humidified CO_2_ incubator. After 24 h, the culture medium was replaced with fresh medium containing the GNPs at increasing concentrations of 0.5 to 32 nM. The concentrations of the GNPs were calculated from their UV-Vis spectra as described previously [47]. As a positive control, cells were treated with 50.0 μM C_2_-Ceramide, which is a known inducer of apoptotic cell death [69]. Untreated cells were used as a negative control. All treatments were done in triplicate. After 24 h, the GNPs were removed and the wells were washed with PBS to ensure complete removal of GNPs. Thereafter, 100 μL of MTT reagent (prepared from 5.0 mg/mL stock solution and diluted with DMEM medium using a dilution factor of 1:10) were added to each well. The plates were incubated again at 37 °C for 4 h. The MTT reagent was then removed and replaced with 100 μL alkaline DMSO to dissolve the purple formazan crystals as recommended by Wang and colleagues [70]. After a 15 min incubation period at 37 °C, the absorbance of the samples was measured at 540 nm using the microtitre plate reader. The absorbance at 630 nm was used as a reference wavelength. The percentage of cell viability was calculated using the following equation:

(1) $$\%\;\mathrm{cell}\;\mathrm{viability}=\frac{\mathrm{sample}\;\mathrm{absorbance}-\mathrm{cell}\;\mathrm{free}\;\mathrm{sample}\;\mathrm{blank}}{\mathrm{negative}\;\mathrm{control}\;\mathrm{absorbance}}$$

### 3.12. Antibacterial Evaluation of the GNPs

Table 6 lists the bacterial strains selected for testing the antibacterial activity of the GNPs in this study. The Alamar blue assay was used to evaluate the inhibition of bacterial growth by both the GNPs and the plant extracts. The test was done according to the manufacturer’s instructions. The bacterial strains were first cultured and maintained on Miller Hinton agar plates. Single colonies were then inoculated into Nutrient broth and incubated at 37 °C with shaking for overnight. The number of bacterial cells was determined and adjusted to 0.5 McFarland using OD_450_ to give final cell concentration of 1–2 × 10^8^ CFU/mL [71]. The cell cultures were further diluted in order to give a final concentration of 5.0 × 10^5^ CFU/mL as recommended by the European committee for Antimicrobial Susceptibility Testing (EUCAST). To determine the MIC values of the tested samples, 50.0 μL of the bacterial broth were mixed, in a 96 microtitre plate, with 50.0 μL of the GNPs (the concentrations of the GNPs varied between 0.5 and 32 nM) or 50.0 μL of the plant extracts (the concentrations of the plant extracts varied between 30.0 and 480 μg/mL). Ampicillin was used as a positive control. Negative controls were also prepared by mixing 50.0 μL of the bacterial culture with 50.0 μL of Nutrient broth. The plates were incubated at 37 °C for 24 h, after which 10.0 μL of the Alamar blue dye were added to each well. The plates were further incubated for a 3 h and then the fluorescence of resorufin was measured using a microtitre plate reader at 544 nm (excitation wavelength) and 590 nm (emission wavelength). To evaluate whether the GNPs and plant extracts interfere with the Alamar blue assay, a sample control was also prepared by mixing 50.0 μL of the GNPs and the plant extracts (all the different concentrations were tested) with 50.0 μL of nutrient broth. The fluorescence of the sample control was subtracted from the sample fluorescence as illustrated in the equation below, which was used to calculate the percentage bacterial growth.

(2) $$\%\;\mathrm{bacterial}\;\mathrm{growth}=\frac{(\mathrm{sample}\;\mathrm{fluorescence}-\mathrm{sample}\;\mathrm{control})-\mathrm{cell}\;\mathrm{free}\;\mathrm{sample}\;\mathrm{blank}}{\mathrm{negative}\;\mathrm{control}\;\mathrm{fluorescence}}\times 100$$

### 3.13. Statistical Analysis

The data presented are means ± SD obtained from at least three independent experiments. Differences between the means were considered to be significant if *p* < 0.05 according to Prism’s two-way ANOVA.

## 4. Conclusions

The study demonstrated an effective and easy methodology for the green synthesis of GNPs from two South African plant extracts, *G. africana* and *H. hemerocallidea*. To the best of our knowledge, this is the first report on GNPs synthesis from these two plants. The GNPs were characterized using different spectroscopic and microscopic techniques such as UV-Vis, DLS analysis, HRTEM, EDX, TGA and FTIR. *G. africana* and *H. hemerocallidea* produced spherical GNPs with an average particle size of 11 ± 1 and 26 ± 6 nm, respectively, as determined by DLS analysis. The FTIR data suggested that the flavonoids of *G. africana* and the glycosides contents of *H. hemerocallidea* might be responsible for the biogenic synthesis of the GNPs. In vitro stability investigation showed that both GNPs, in particular Hypoxis-GNPs, are stable when incubated with different biological buffers and the culture media. Hypoxis-GNPs showed a higher antibacterial effect compared to Galenia-GNPs against the bacterial strains tested in this study. Both GNPs were found to be non-toxic against a non-cancerous human fibroblast cell line suggesting that it may be safe to use these GNPs in wound dressings for the prevention of wound infections. However, more cytotoxic assays should be carried out to fully determine their toxicity. Additionally, a wider panel of bacterial strains that are known to cause skin infections should be investigated.

## Figures and Tables

**Table 1:** Average diameter of Galenia-GNPs and Hypoxis-GNPs obtained from DLS analysis.

GNPs	Small-Particle Peaks Average Diameter (nm)	Large-Particle Peaks Average Diameter (nm)	Z-Average Diameter (nm)
Galenia-GNPs	1.9 ± 1.1	44 ± 29	11 ± 1
Hypoxis-GNPs	2.3 ± 1.6	51 ± 34	26 ± 6

**Table 2:** Shifts of the FTIR spectra bands (cm^−1^) of the major peaks of *H. hemerocallidea* and *G. africana* aqueous extracts and their respective GNPs.

*G. africana*				*H. hemerocallidea*			
Aqueous Extract	Galenia-GNPs	Shift Value *	Possible Functional Groups	Aqueous Extract	Hypoxis-GNPs	Shift Value *	Possible Functional Groups
3403	3428	−25	O–H Alcohols	3390	3420	−30	O–H Alcohols
2931	2924	+7	C–H Alkanes	2924	2923	+1	C–H Alkanes
1384	1384	0	–CH_3_ Alkanes	1438	1402	+36	C=C Aromatics
1320	1329	−9	O–H Alcohols, Phenols	1249	1267	−18	C–O Aromatic esters, Ethers, Carboxylic acids
775	758	+17	C–Cl Alkanes C–H Benzenes	1072	1067	−5	C–O–C

* The shift values were calculated by subtracting the peak transmittence of GNPs from the peak transmittance of the extract.

**Table 3:** Average particle size of Galenia-GNPs and Hypoxis-GNPs as obtained from the Zetasizer, HRTEM and UV-Vis spectra.

Type of GNPs	Average Size (nm)		
	Zetasizer	HRTEM	UV-Vis
Galenia-GNPs	11 ± 1	9 ± 2	10
Hypoxis-GNPs	26 ± 6	27 ± 6	18
Difference in size *	+15	+18	+8

* Difference was calculated as follow: (average size of Hypoxis-GNPs) − (average size of Galenia-GNPs).

**Table 4:** Weight (expressed as percentage) of *H. hemerocallidea* and *G. africana* extracts and their respective GNPs at different temperatures as obtained from TGA.

Sample	Weight % at 100 °C	Weight % at 400 °C	Weight % at 800 °C
Hypoxis-GNPs	100%	97.5%	91.8%
Galenia-GNPs	100%	96.6%	87.7%
*H. hemerocallidea* extract	96.8%	52%	34%
*G. africana* extract	99.2%	60%	39%

**Table 5:** The MIC values of the GNPs, the aqueous extracts and Ampicillin on the tested bacterial strains. The viability recorded at each MIC of the GNPs are written in brackets.

Sample Tested	Bacterial Strains			
	*S. aureus*	*E. coli*	*S. epidermidis*	*P. aeruginosa*
Hypoxis-GNPs (nM)	32 (43 ± 5%) *	32 (16 ± 1%) ***	32 (20 ± 1%) ***	32 (10 ± 1%) ***
Galenia-GNPs (nM)	>32	>32	>32	32 (35 ± 5%) **
Citrate GNPs (nM)	>32	>32	>32	>32
*H. hemerocallidea* extract (mg/mL)	>0.48	>0.48	>0.48	>0.48
*G. africana* extract (mg/mL)	>0.48	>0.48	>0.48	>0.48
Ampicillin (mg/mL)	0.004	0.002	0.0005	2.0

*** Statistical significance (*p* < 0.001) compared to negative control, ** Statistical significance (*p* < 0.01) compared to negative control, * Statistical significance (*p* < 0.05) compared to negative control.

**Table 6:** List of bacterial strains used in the antibacterial assay.

Bacterial Strains	ATCC Number	Gram Reaction
*E. coli*	25,922	Gram-negative
*P. aeruginosa*	27,853	Gram-negative
*S. aureus*	29,213	Gram-positive
*S. epidermidis*	12,228	Gram-positive
